# Self-Rated Health and Burnout Dimensions Among Prehospital Emergency Medical Services Physicians: A Cross-Sectional Study

**DOI:** 10.3390/bs16101747

**Published:** 2026-09-24

**Authors:** Veroslava V. Stanković, Dragana P. Jović, Nataša K. Rančić, Ivana D. Milivojčević Bevc, Goran Z. Čolaković, Ivana M. Stefanović, Danijela M. Tasić-Uroš

**Affiliations:** 1 The College of Health Sciences, Academy of Applied Studies Belgrade, Cara Dušana 254, 11080 Belgrade, Serbia; 2 Institute of Public Health of Serbia “Dr Milan Jovanović Batut”, Dr Subotića Starijeg 5, 11000 Belgrade, Serbia; drdraganajovic@gmail.com; 3 Faculty of Medicine, University of Niš, Bulevar Dr. Zorana Đinđića 81, 18000 Niš, Serbia; drnatasarancic@gmail.com; 4 Institute of Public Health Niš, Bulevar Dr. Zorana Đinđića 50, 18000 Niš, Serbia; 5 Institute for Emergency Medicine Belgrade, Franše d’Eperea 5, 11000 Belgrade, Serbia; ivana.bevc@yahoo.com (I.D.M.B.); drcolakovic@beograd194.rs (G.Z.Č.); ivanastf@gmail.com (I.M.S.); tasicurosdanijela@gmail.com (D.M.T.-U.)

**Keywords:** self-rated health, occupational burnout, emotional exhaustion, depersonalisation, reduced personal accomplishment, prehospital emergency medical services, physicians

## Abstract

This study examined associations between self-rated health (SRH) and burnout dimensions among prehospital emergency physicians. This cross-sectional study included a convenience sample of 151 physicians from one emergency medical service in Belgrade, Serbia. Burnout was assessed using the Maslach Burnout Inventory–Human Services Survey and SRH using a single SF-36 general-health item. Ordinal models examined three ordered SRH categories (excellent/very good, good, and fair/poor), with burnout dimensions entered separately and jointly, adjusting for age, sex, professional qualification, and chronic disease. Binary sensitivity analyses compared fair/poor with excellent/very good/good health. Higher emotional exhaustion was associated with less favourable SRH in the separate adjusted ordinal model (OR per 10-point increase: 1.31; 95% CI 1.04–1.64). After mutual adjustment for burnout dimensions, the estimate remained similar but was less precise (OR 1.30; 95% CI 0.96–1.75). Evidence was also inconclusive in the adjusted binary analysis. Depersonalisation and reduced personal accomplishment showed no clear adjusted associations with SRH. Unadjusted associations of shift work and daily working hours with SRH were inconclusive. Emotional exhaustion may be associated with physicians’ perceived health, although the evidence varied across models. The cross-sectional design precludes conclusions about direction or causality; longitudinal studies are needed.

## 1. Introduction

Emergency medicine is a demanding clinical environment characterised by unpredictable workloads, life-threatening situations, rapid decision-making, night work, and limited opportunities for recovery. Systematic reviews and meta-analyses have documented a substantial burden of burnout among emergency department staff (Alanazy & Alruwaili, 2023; Gualano et al., 2021), emergency physicians (Zhang et al., 2020), and paramedics (Reardon et al., 2020). Physicians working in prehospital emergency medical services face additional challenges associated with providing care outside hospital settings, often with limited information and resources and in close contact with patients and families during crises.

The World Health Organization identifies burnout in the 11th Revision of the International Classification of Diseases (ICD-11) as an occupational phenomenon associated with persistent, inadequately managed work-related stress (World Health Organization, 2019). The Maslach framework distinguishes three dimensions: emotional exhaustion (EE), depersonalisation (DP), and personal accomplishment (PA). Emotional exhaustion refers to depleted emotional and energetic resources, whereas depersonalisation involves detached or impersonal responses towards recipients of care. Personal accomplishment concerns feelings of professional competence and achievement, with diminished feelings indicating greater burnout (Maslach et al., 2001). These dimensions describe different aspects of occupational experience, providing a rationale for examining their associations with perceived health individually and jointly.

Research on burnout has considered both organisational and individual factors (Vukmirović & Milić, 2023). These include workload, autonomy, leadership, social support, organisational climate, and work–family conflict (Amiri et al., 2024; Ghaseminejad et al., 2025; Nagle et al., 2024; Shoman et al., 2021). However, the definition of burnout and the evidence concerning its causes remain debated (Bianchi & Schonfeld, 2023). A meta-analysis of longitudinal studies examining predictors of exhaustion reported predominantly weak associations and generally low-quality evidence (Shoman et al., 2022). This uncertainty supports cautious interpretation of potential determinants and a clear distinction between observed associations and causal relationships.

Burnout has been associated with psychological, physical, and occupational difficulties, including depressive symptoms, sleep disturbance, somatic complaints, sickness absence, and presenteeism (Salvagioni et al., 2017). Reviews have also addressed the implications of burnout for healthcare workers and care delivery (De Hert, 2020), associations with patient safety across healthcare staff (Hall et al., 2016), and associations with patient safety, satisfaction, and quality of care specifically among nurses (Li et al., 2024). Among prehospital emergency physicians, burnout has been associated with turnover intention (Feng et al., 2022), while research among paramedics and emergency medical technicians has also linked burnout to sickness absence and intentions to leave the profession (Powell et al., 2025). These findings support examining burnout alongside indicators of physicians’ own health.

Self-rated health (SRH) captures an individual’s overall appraisal of health. Early longitudinal research examined such self-assessments among older adults (Maddox & Douglass, 1973). A single global rating can integrate diagnosed illness, symptoms, functional limitations, and subjective experiences of physical and psychological well-being (Jylhä, 2009). It therefore provides a complementary perspective to measures focused specifically on occupational experiences. Because emotional exhaustion, depersonalisation, and personal accomplishment concern different aspects of burnout, their relationships with this overall health appraisal may also differ. Examining the dimensions separately and together can help clarify whether their associations with SRH persist when their overlap is taken into account.

Previous research supports examining SRH in relation to occupational well-being. In a general adult population, longitudinal findings suggest bidirectional associations between suboptimal SRH and categorically defined burnout, disturbed sleep, and somatic symptoms (Nordin et al., 2024). A Finnish physician survey examined burnout and perceived health among psychiatrists and child psychiatrists (Korkeila et al., 2003), while a study of Japanese occupational physicians linked job stress to SRH (Shimizu & Nagata, 2005). Among emergency physicians, favourable SRH has been associated with better sleep, fewer night shifts, lower workload, absence of workplace violence, and a more favourable effort–reward balance (Peng et al., 2023). These findings also provide a rationale for considering work schedules when examining physicians’ SRH.

Studies using the SF-36 have reported associations between burnout and poorer health-related quality of life among emergency physicians (Sarwar et al., 2023) and private security employees (Rancic et al., 2024). A systematic review of studies among nurses also identified associations between burnout and quality of life, assessed using several different instruments (Khatatbeh et al., 2022). Such findings provide complementary evidence concerning health-related quality of life, although these broader measures are not equivalent to a single global SRH rating. Examining global SRH specifically may therefore add to the understanding of how physicians’ overall perceptions of health relate to individual burnout dimensions.

Research from Serbia has documented burnout among healthcare professionals (Safiye et al., 2022, 2025). Further investigation is warranted to clarify how individual burnout dimensions relate to physicians’ health perceptions within prehospital emergency medical services. The Institute for Emergency Medicine Belgrade provides a setting for examining these relationships in a large urban service, while accounting for demographic, professional, and health characteristics.

The primary objective was to examine the associations of self-rated health (SRH) with emotional exhaustion, depersonalisation, and personal accomplishment among physicians working in a large urban prehospital emergency medical service. The secondary objective was to examine the associations of shift work and average daily working hours with SRH.

## 2. Materials and Methods

### 2.1. Study Design, Setting, and Ethics

This cross-sectional study was conducted at the Institute for Emergency Medicine Belgrade, Serbia, from April 2024 to January 2025.

The Institute for Emergency Medicine Belgrade provides prehospital emergency care across 11 urban municipalities (Institute for Emergency Medicine Belgrade, n.d.-d). It operates continuously through one central facility and 13 emergency medical stations, with 25 physician teams scheduled per 12 h shift; field teams and adult outpatient emergency units are described in its service overview (Institute for Emergency Medicine Belgrade, n.d.-a). Its organisation also includes emergency-call reception, triage, and dispatch (Institute for Emergency Medicine Belgrade, n.d.-c), paediatric emergency services (Institute for Emergency Medicine Belgrade, n.d.-b), and staff education (Institute for Emergency Medicine Belgrade, n.d.-d). The service operates across geographically dispersed locations, with continuous readiness to provide emergency care to critically ill or injured patients.

Participation was voluntary, and the confidentiality of the study data was maintained. Physicians approached for recruitment received information about the study aims and procedures, confidentiality, and use of the data exclusively for research purposes. All participants included in the final analytical sample provided written informed consent. Institutional permission was obtained from the Director of the Institute for Emergency Medicine Belgrade.

The study was conducted in accordance with the ethical principles of the Declaration of Helsinki (World Medical Association, 2013). The study protocol was approved by the Ethics Committee of the Institute for Emergency Medicine Belgrade, before recruitment began (approval no. 752/1; approval date: 30 January 2024).

### 2.2. Participants and Sampling

At the time of the study, the Institute for Emergency Medicine Belgrade employed 239 physicians, including 221 permanent and 18 fixed-term employees. The source population comprised the 221 permanently employed physicians; fixed-term employees were outside the target population.

Inclusion criteria

Eligible participants were physicians who were citizens of the Republic of Serbia, were employed full-time at the Institute on a permanent basis, and held a valid professional licence. Participation was voluntary and required written informed consent.

Exclusion criteria and nonparticipation

Physicians who did not meet the employment, citizenship, or professional-licensure requirements were ineligible, whereas otherwise eligible physicians who declined participation were not enrolled. Neither long-term sick leave nor recent major psychophysical trauma was applied as an exclusion criterion.

Institutional tenure and shift-work status

Total years of medical practice, years of employment at the Institute, and shift-work status were recorded separately. Institutional tenure referred specifically to years worked at the Institute. Neither a minimum institutional tenure nor shift work was an eligibility requirement.

Recruitment and sampling

For a finite population of 221 physicians, the minimum sample size was calculated as 141, assuming a 95% confidence level, a 5% margin of error, and an anticipated population proportion of 50%. This calculation informed the advance preparation of 200 paper questionnaires. Recruitment followed a non-probability convenience-sampling approach: questionnaires were distributed across organisational units and work shifts to available physicians who were willing to participate. No formal probability-sampling procedure was used; therefore, the achieved sample does not have a design-based 5% margin of error.

The 200 questionnaires were distributed to 150 women and 50 men. The remaining 21 permanently employed physicians did not receive a questionnaire; individual reasons for non-distribution were not recorded.

Questionnaire screening and analytical sample

Of the 200 distributed questionnaires, 173 were returned, corresponding to a questionnaire return rate of 86.5%, and 27 were not returned.

Returned questionnaires were screened for completeness and the presence of the required signed informed consent form. A questionnaire was considered incomplete if it contained at least one unanswered question anywhere in the full questionnaire, regardless of whether that question concerned a variable used in the analyses reported in this article. Questionnaires were excluded if they were incomplete, lacked signed informed consent, or met both criteria. These exclusions occurred during screening of returned questionnaires and were distinguished from ineligibility or nonparticipation during recruitment.

Following screening, 22 returned questionnaires were excluded: 15 because of incomplete responses only, two because of missing signed informed consent only, and five because of both reasons. These mutually exclusive categories accounted for all 22 exclusions, with each questionnaire counted once.

The final analytical sample comprised 151 physicians, including 131 shift workers and 20 non-shift workers. This represented 75.5% of the distributed questionnaires and 68.3% of the source population of 221 permanently employed physicians. The 21 physicians who did not receive a questionnaire, the 27 who did not return one, and the 22 whose returned questionnaires were excluded constituted distinct groups.

### 2.3. Measures

Data were collected using a questionnaire comprising a study-specific section on sociodemographic, occupational, and health characteristics, the Maslach Burnout Inventory–Human Services Survey (MBI-HSS), and the SF-36 Health Survey. All responses were treated as confidential. The present analyses used sex, age, professional qualification, total duration of medical practice, duration of employment at the Institute for Emergency Medicine Belgrade, shift-work status, average daily working hours, self-rated household material status, and the presence of chronic disease.

Age and the two measures of professional experience were recorded in years. Professional qualification was grouped as a basic academic degree or specialisation/subspecialisation. Shift work and chronic disease were recorded as yes/no variables. Average daily working hours were analysed in three categories: 8 h, >8–12 h, and >12 h. Household material status was grouped as good/very good, average, or poor/very poor.

The MBI-HSS is a 22-item instrument comprising three subscales: emotional exhaustion (EE; nine items), depersonalisation (DP; five items), and personal accomplishment (PA; eight items) (Maslach et al., 2001, 2012). Items are rated on a seven-point frequency scale ranging from 0 (never) to 6 (every day). The Croatian-language version of the MBI-HSS supplied by SINAPSA EDITION was administered. Scoring followed the Croatian-language manual translated by L. Arambašić (Maslach et al., 2012).

EE and DP scores were obtained by summing their nine and five items, respectively, giving theoretical ranges of 0–54 and 0–30. The original PA score is the sum of eight items, with a theoretical range of 0–48. Higher EE and DP scores and lower PA scores indicate more pronounced burnout-related difficulties in the respective dimensions (Maslach et al., 2012).

For the present analyses, PA items were reverse-scored before summation to obtain reduced personal accomplishment (RPA). For each of the eight PA items (items 4, 7, 9, 12, 17, 18, 19, and 21), the original response x on the 0–6 scale was transformed to 6 − x. Thus, RPA = 48 − PA, with a theoretical range of 0–48. EE and DP items were not reverse-scored. Higher EE, DP, and RPA scores therefore indicate greater burnout-related difficulties. The three dimensions were analysed as continuous scores, and no categorical definition of burnout was applied. Internal consistency was assessed using Cronbach’s α for each subscale.

Permission to use the MBI-HSS questionnaire and its scoring key was obtained directly from SINAPSA EDITION (licence no. 2/2018, dated 9 May 2018). The use of the MBI-HSS in healthcare settings has been documented in a scoping review (Soares et al., 2023), and its psychometric properties have been examined among Serbian anaesthesiologists (Matejić et al., 2015) and Spanish emergency-care personnel (Forné & Yuguero, 2022).

The SF-36 is a generic measure of health-related quality of life with established psychometric properties (Brazier et al., 1992; McHorney et al., 1994; Ware & Sherbourne, 1992). In the present study, the Serbian-language version of the SF-36, previously validated in patients with arterial hypertension (Nikolic et al., 2023), was administered. Although the full questionnaire was administered, self-rated health (SRH) was assessed exclusively using its global general-health item: “In general, would you say your health is excellent, very good, good, fair, or poor?” (Ware & Sherbourne, 1992). This item provides a concise appraisal of perceived health that can be considered independently of the domain scores. A single overall SF-36 total score is not supported by the instrument’s original scoring framework (Lins & Carvalho, 2016). In the present study, neither a total SF-36 score nor the multi-item general-health subscale was used as the SRH outcome.

SRH was the outcome variable. For the primary analyses, responses were grouped into three ordered categories: excellent/very good (*n* = 84), good (*n* = 30), and fair/poor (*n* = 37), ordered from more to less favourable health. Excellent and very good responses were combined to focus the analysis on broader differences in perceived health rather than distinctions between the two most favourable response options. The combined fair/poor category included the six participants who reported poor health. This grouping retained good health as a distinct intermediate category and combined the six poor-health responses with the adjacent fair category to reduce sparsity at the less favourable end of the scale while preserving an ordered outcome. Frequencies for all five original responses are reported in Table 1, and their mapping to the analytical categories is shown in Table A1.

For the sensitivity analysis, SRH was dichotomised as fair/poor (*n* = 37) versus excellent/very good/good (*n* = 114), with fair/poor health as the outcome category of interest. This binary grouping is also presented in Table A1.

### 2.4. Statistical Analysis

Statistical analyses were performed using IBM SPSS Statistics for Windows, version 26.0 (IBM Corp., Armonk, NY, USA). Categorical variables were summarised as frequencies and percentages. Continuous variables were presented as medians with interquartile ranges and observed ranges. Means and standard deviations were additionally reported for the three Maslach Burnout Inventory–Human Services Survey (MBI-HSS) subscale scores. The internal consistency reliability of each MBI-HSS subscale was assessed using Cronbach’s alpha.

For the primary analyses, self-rated health was grouped into three ordered categories: excellent/very good, good and fair/poor. Differences in emotional exhaustion, depersonalisation and reduced personal accomplishment scores across these categories were examined using the Kruskal–Wallis test.

Ordinal logistic regression with a cumulative logit link was used to examine associations between the three burnout dimensions and self-rated health. The outcome categories were ordered from excellent/very good to good and fair/poor; consequently, odds ratios greater than 1 indicated higher odds of being in a less favourable self-rated health category. Emotional exhaustion, depersonalisation and reduced personal accomplishment were analysed as continuous scores, and the corresponding odds ratios were expressed per 10-point increase.

Each burnout dimension was first examined in a separate unadjusted model and subsequently in a separate multivariable model adjusted for age, sex, professional qualification and the presence of chronic disease. A mutually adjusted model was then fitted in which all three burnout dimensions were entered simultaneously together with the same covariates. Age was entered as a continuous variable. Female sex, a basic academic degree and the absence of chronic disease were used as the reference categories for the categorical covariates. The covariates were selected a priori on the basis of their substantive relevance to self-rated health and the need to maintain model parsimony given the sample size. Age and sex were included for demographic adjustment, professional qualification to represent professional background, and chronic disease to account for a measured aspect of health status.

The proportional-odds assumption was evaluated using the test of parallel lines for each ordinal logistic regression model. Overall model significance was assessed using likelihood-ratio chi-square tests, whereas individual regression coefficients were evaluated using Wald tests. Multicollinearity among the predictors included in the mutually adjusted model was assessed using tolerance and variance inflation factor values. Tolerance values below 0.20 and variance inflation factor values above 5 were regarded as indicating potentially meaningful multicollinearity.

As a sensitivity analysis, self-rated health was dichotomised as fair/poor versus excellent/very good/good. Separate binary logistic regression models were fitted for each burnout dimension. Both unadjusted models and models adjusted for age, sex, professional qualification and chronic disease were estimated. Odds ratios for the burnout dimensions were again expressed per 10-point increase in the respective subscale score.

Associations of shift-work status and average daily working hours with the three-category self-rated health outcome were examined in separate unadjusted ordinal logistic regression models. Physicians who did not work shifts and those working 8 h per day were used as the respective reference categories. The proportional-odds assumption was also evaluated for these models.

Following the questionnaire screening described in Section 2.2, there were no missing values in the variables analysed among the 151 included physicians. All analyses used this final sample. Odds ratios are reported with 95% confidence intervals. All statistical tests were two-sided, and *p* values below 0.05 were considered statistically significant.

## 3. Results

### 3.1. Participant Characteristics

Women comprised 74.8% of the study population. The median age in the sample was 46 years (IQR 39–52), and approximately half of the physicians were aged 45–59 years (51.0%). Most participants had completed a specialisation or subspecialisation and had considerable professional experience, both in medical practice overall and at their current health institution. Shift work was the prevailing working arrangement, and extended daily working hours were common. Household material status was most frequently rated as good or very good. Approximately one-third of the physicians reported at least one chronic disease. More than half rated their health as excellent or very good (55.6%), whereas almost one-quarter were classified in the combined fair/poor self-rated health category (24.5%) used in the subsequent analyses (Table 1).

### 3.2. Burnout Dimensions and Internal Consistency

Scores on the three dimensions of the Maslach Burnout Inventory–Human Services Survey (MBI-HSS) varied considerably across participants. The median emotional exhaustion score was 22 (IQR 11–36; observed range 0–51). The corresponding median scores were 7 for depersonalisation (IQR 1–11; observed range 0–23) and 12 for reduced personal accomplishment (IQR 7–18; observed range 0–30).

Cronbach’s alpha indicated excellent internal consistency reliability for the emotional exhaustion subscale (α = 0.940) and good internal consistency reliability for the depersonalisation subscale (α = 0.825). The reduced personal accomplishment subscale demonstrated lower internal consistency reliability, with its coefficient falling below the conventional threshold of 0.70 (α = 0.655) (Table 2).

### 3.3. Burnout Dimensions According to Self-Rated Health

Scores on the emotional exhaustion subscale differed significantly across the three self-rated health categories (overall *p* = 0.005). The median emotional exhaustion score increased from 17 (IQR 9–33.5) among physicians reporting excellent or very good health to 30 (IQR 14.75–38) among those reporting good health and 33 (IQR 14–43) among those reporting fair or poor health.

By contrast, scores on the depersonalisation and reduced personal accomplishment subscales did not differ significantly across the self-rated health categories (overall *p* = 0.272 and *p* = 0.507, respectively). The median depersonalisation subscale scores were 6, 9 and 9 in the excellent/very good, good and fair/poor health categories, respectively. The corresponding median reduced personal accomplishment subscale scores were broadly similar at 12, 12.5 and 12 (Table 3).

### 3.4. Ordinal Logistic Regression Analysis

In the unadjusted ordinal logistic regression models, higher scores on the emotional exhaustion and depersonalisation subscales were associated with less favourable self-rated health. For each 10-point increase in the emotional exhaustion score, the odds of being in a less favourable self-rated health category increased by 44% (OR 1.44, 95% CI 1.16–1.78; *p* = 0.001). A 10-point increase in the depersonalisation score was associated with 59% higher odds of less favourable self-rated health (OR 1.59, 95% CI 1.03–2.45; *p* = 0.037). The reduced personal accomplishment score was not significantly associated with self-rated health (OR 1.34, 95% CI 0.89–2.03; *p* = 0.164).

After adjustment for age, sex, professional qualification and chronic disease, the emotional exhaustion score remained significantly associated with less favourable self-rated health. Each 10-point increase was associated with 31% higher odds of belonging to a less favourable self-rated health category (adjusted OR 1.31, 95% CI 1.04–1.64; *p* = 0.020). The association with the depersonalisation score was attenuated and was no longer statistically significant (adjusted OR 1.44, 95% CI 0.91–2.29; *p* = 0.118). No statistically significant association was found between the reduced personal accomplishment score and self-rated health in the adjusted model (adjusted OR 1.12, 95% CI 0.72–1.76; *p* = 0.609).

When all three burnout dimensions were entered simultaneously together with the covariates, no statistically significant association was found for any individual burnout dimension. The estimated association for emotional exhaustion remained essentially unchanged compared with the separate adjusted model (OR 1.31 versus 1.30 in the mutually adjusted model). However, the 95% confidence interval widened from 1.04–1.64 to 0.96–1.75, indicating reduced precision, and the association was no longer statistically significant in the mutually adjusted model (*p* = 0.087). The corresponding estimates were 1.03 (95% CI 0.55–1.94; *p* = 0.930) for depersonalisation and 0.98 (95% CI 0.61–1.59; *p* = 0.942) for reduced personal accomplishment (Table 4).

The overall likelihood-ratio tests indicated statistically significant model improvement for the unadjusted emotional exhaustion and depersonalisation models, whereas the unadjusted reduced personal accomplishment model was not statistically significant (χ^2^(1) = 11.389, *p* = 0.001; χ^2^(1) = 4.325, *p* = 0.038; and χ^2^(1) = 1.961, *p* = 0.161, respectively). All three separately adjusted models were statistically significant overall: emotional exhaustion, χ^2^(5) = 29.226, *p* < 0.001; depersonalisation, χ^2^(5) = 26.368, *p* < 0.001; and reduced personal accomplishment, χ^2^(5) = 24.157, *p* < 0.001. The mutually adjusted model was also statistically significant overall (likelihood-ratio χ^2^(7) = 29.236, *p* < 0.001). Overall model significance does not imply that each individual predictor is statistically significant.

In the mutually adjusted model, no potentially meaningful multicollinearity was identified. Tolerance values ranged from 0.518 to 0.893, and variance inflation factor values ranged from 1.120 to 1.931 (Table 5).

### 3.5. Sensitivity Analysis Using Binary Self-Rated Health

The sensitivity analysis in which fair/poor self-rated health was treated as a binary outcome yielded estimates that were generally consistent in direction with those from the primary ordinal analysis. In the unadjusted binary logistic regression model, each 10-point increase in the emotional exhaustion score was associated with 41% higher odds of fair or poor self-rated health (OR 1.41, 95% CI 1.09–1.82; *p* = 0.008). After adjustment for age, sex, professional qualification and chronic disease, the association was attenuated and was no longer statistically significant (adjusted OR 1.24, 95% CI 0.95–1.63; *p* = 0.118).

The depersonalisation score was not significantly associated with fair or poor self-rated health in either the unadjusted model (OR 1.63, 95% CI 0.98–2.73; *p* = 0.060) or the adjusted model (adjusted OR 1.51, 95% CI 0.86–2.63; *p* = 0.149). Similarly, no statistically significant associations were found for the reduced personal accomplishment score in the unadjusted (OR 1.40, 95% CI 0.86–2.30; *p* = 0.177) or adjusted model (adjusted OR 1.22, 95% CI 0.70–2.11; *p* = 0.483) (Table 6). A comparison of adjusted ordinal and binary estimates is provided in Table A2.

### 3.6. Self-Rated Health According to Work Schedule

Self-rated health did not differ significantly according to shift-work status. Among physicians working shifts, 56.5% reported excellent or very good health, 19.8% reported good health and 23.7% reported fair or poor health. The corresponding proportions among physicians who did not work shifts were 50.0%, 20.0% and 30.0%, respectively. No statistically significant association was found between shift work and less favourable self-rated health (OR 0.75, 95% CI 0.31–1.83; *p* = 0.532).

No statistically significant association was found between average daily working hours and self-rated health (overall model *p* = 0.991). With physicians working 8 h per day as the reference group, the OR point estimates were close to 1 for those working >8–12 h (OR 0.94, 95% CI 0.35–2.52; *p* = 0.898) and those working >12 h (OR 0.94, 95% CI 0.31–2.85; *p* = 0.906). However, the wide confidence intervals indicated limited precision and were compatible with both lower and higher odds of less favourable self-rated health (Table 7). The corresponding descriptive distribution under the binary SRH grouping is presented in Table A3.

The tests of parallel lines were non-significant for all nine ordinal logistic regression models, with *p* values ranging from 0.105 to 0.912. Thus, no evidence of departure from the proportional-odds assumption was found for the separate or mutually adjusted burnout models or for the models examining shift work and average daily working hours (Table 8).

## 4. Discussion

Emotional exhaustion was associated with less favourable SRH in the separate adjusted ordinal model, but the evidence was less conclusive in the mutually adjusted ordinal and adjusted binary models. This pattern suggests a possible association between exhaustion and perceived health whose independence from other burnout dimensions and consistency across outcome definitions remain uncertain.

Exhaustion may be particularly relevant to global health appraisals because these can encompass symptoms, mood and functioning (Maslach et al., 2001; Jylhä, 2009), although this explanation was not tested here. Research among Finnish physicians and Swedish healthcare workers provides related evidence on burnout and perceived health (Korkeila et al., 2003; Peterson et al., 2008). Comparisons with studies of emergency physicians, security employees and nurses require caution because they examined broader quality-of-life outcomes (Sarwar et al., 2023; Rancic et al., 2024; Khatatbeh et al., 2022). Their findings contextualise the present association but do not directly replicate an analysis based on a single SRH item.

Longitudinal findings in a general adult population suggest reciprocal relationships between suboptimal SRH and categorically defined burnout, disturbed sleep and somatic symptoms (Nordin et al., 2024). Exhaustion may contribute to poorer health perceptions, while existing health difficulties may reduce the resources available for coping with work. These possible pathways warrant investigation in prehospital physicians using comparable measures.

For each 10-point increase in emotional exhaustion, the adjusted OR was 1.31 (95% CI 1.04–1.64) in the separate ordinal model and 1.30 (95% CI 0.96–1.75) after depersonalisation and reduced personal accomplishment were added. The change in statistical significance therefore accompanied a wider confidence interval, with little change in the estimated association. The mutually adjusted model estimates the association of each burnout dimension with SRH conditional on the other two dimensions and the measured covariates. Correlation among predictors can affect the precision of conditional estimates, but a change in statistical significance alone does not establish problematic multicollinearity (O’Brien, 2007). In the present study, VIF values ranged from 1.120 to 1.931 and tolerance values from 0.518 to 0.893, providing no indication of problematic multicollinearity under the prespecified criteria (Table 5).

In the adjusted binary analysis, the emotional-exhaustion point estimate remained above 1 but was imprecise (OR 1.24, 95% CI 0.95–1.63; *p* = 0.118). Combining “good” with excellent and very good health changes the outcome contrast and discards information retained by the ordinal model. This may contribute to differences in precision. Statistical significance in one model and its absence in another do not themselves demonstrate a difference between associations (Gelman & Stern, 2006).

Depersonalisation showed no clear adjusted association with SRH, and evidence for reduced personal accomplishment was also inconclusive. Interpersonal distancing and diminished perceived competence may be less directly reflected in a global health rating than exhaustion (Maslach et al., 2001). The MBI-HSS asks about the frequency of occupational experiences, whereas the SRH item concerns health in general (Maslach et al., 2012; Ware & Sherbourne, 1992). Differences in item content and the time frames respondents had in mind may also have contributed to the observed pattern, although these possibilities were not examined in this study. The findings do not establish that the associations differ between dimensions.

Neither shift work nor average daily working hours showed a clear association with SRH in the separate unadjusted ordinal analyses. These models did not account for demographic or health differences between work-schedule groups. Most participants worked shifts, and the reference groups for shift work and working hours were relatively small, contributing to limited precision. The broad schedule categories also provide little information about night-shift frequency, consecutive shifts, workload intensity, or recovery opportunities. Research among emergency physicians has linked more detailed measures of sleep, night work, workload, and workplace violence to SRH (Peng et al., 2023). A separate study examined perceived work demands and emergency department crowding in relation to objective stress across physicians’ shifts (Britt et al., 2024). Among EMS providers, more frequent night shifts have also been associated with burnout, although that outcome differs from SRH (Stuby et al., 2026). The present findings therefore do not establish that different work schedules are equivalent in their health implications.

### 4.1. Strengths and Limitations

A principal strength is the focus on physicians within a clearly defined urban prehospital service. This adds a specific occupational perspective to research among Belgrade emergency-service employees (Baić, 2017), Serbian healthcare workers (Safiye et al., 2022, 2025), and hospital doctors and family physicians in the region (Stanetić et al., 2016). Burnout was examined dimensionally using an established instrument, and the emotional-exhaustion and depersonalisation subscales had strong internal consistency. Reporting all original SRH responses, retaining an intermediate category in the primary analysis, and comparing separate, mutually adjusted, and binary models made the analytical choices transparent. All analyses used the same sample of 151 physicians.

Several considerations are relevant to the interpretation of these findings. The cross-sectional design allows associations to be examined but does not establish temporal order or causality. The use of self-reported measures may introduce reporting and common-method bias. Adjustment for age, sex, professional qualification and chronic disease addressed selected relevant factors, although residual confounding remains possible. Chronic disease was recorded as present or absent, and the models did not include disease severity, sleep, psychological symptoms or detailed occupational exposures.

Internal consistency was lower for reduced personal accomplishment (Cronbach’s α = 0.655), which should be considered when interpreting findings for this dimension. The SRH item provided a global assessment of perceived health without distinguishing individual health domains. Grouping adjacent responses accommodated small category sizes at the expense of some detail, while retaining three ordered categories in the primary analysis and allowing comparison with a binary sensitivity analysis. Some estimates had wide confidence intervals, particularly in the mutually adjusted and work-schedule models. The work-schedule analyses used unadjusted models and broad categories.

The analytical sample included 68.3% of the institution’s permanently employed physicians, providing substantial coverage of this workforce. Recruitment nevertheless depended on availability and voluntary participation. Physicians experiencing greater exhaustion may have been less available or willing to participate, although this possibility could not be assessed using the available data. Comparative health and burnout data were unavailable for nonparticipants and respondents excluded during screening. Generalisability should therefore be considered in the context of a single urban prehospital service and a predominantly female sample.

### 4.2. Implications and Future Research

The study supports further investigation of SRH alongside individual burnout dimensions, particularly emotional exhaustion. It does not establish screening thresholds, predictive performance, or the benefit of using these measures for occupational monitoring. Although SRH has prognostic value in other populations (Idler & Benyamini, 1997; Miilunpalo et al., 1997), this cannot be assumed for a proposed monitoring approach in prehospital physicians. Longitudinal, multicentre studies should assess changes in health and burnout, retain ordered SRH responses where feasible, and collect more detailed information on sleep, recovery, psychological symptoms, disease burden, and work schedules. Prespecified models and independent validation would help clarify which associations are reproducible and whether the measures add useful information.

Future intervention research should evaluate organisational changes alongside individual support, drawing on existing work on clinician burnout and workplace well-being (Cohen et al., 2023; West et al., 2016, 2018). Staffing, scheduling, recovery opportunities, team communication, and confidential access to support are relevant areas for evaluation. Broader research links physician burnout with career disengagement and poorer quality of patient care (Hodkinson et al., 2022), burnout among healthcare staff with patient safety outcomes (Hall et al., 2016), and physician burnout with reported medical errors (Tawfik et al., 2018). These outcomes should be measured directly in future studies. The present findings do not establish intervention effectiveness or consequences for patient safety.

## 5. Conclusions

In this single-service sample, emotional exhaustion may be associated with physicians’ perceived health, although the strength of evidence varied across analyses. The cross-sectional design precludes conclusions about direction or causality. Longitudinal, multicentre studies are needed to clarify this relationship, assess occupational exposures in greater detail, and examine its potential relevance for occupational health.

## Figures and Tables

**Table 1:** Sociodemographic, occupational, and health characteristics of the study participants (*N* = 151).

Variables	*n* (%)	Median (IQR)	Range
**Sex**			
Female	113 (74.8)	—	—
Male	38 (25.2)	—	—
Age, years	—	46 (39–52)	25–63
**Age group, years**			
<30	18 (11.9)	—	—
30–44	48 (31.8)	—	—
45–59	77 (51.0)	—	—
≥60	8 (5.3)	—	—
**Professional qualification**			
Basic academic degree	52 (34.4)	—	—
Specialisation or subspecialisation	99 (65.6)	—	—
Total duration of medical practice, years	—	16 (10–24)	0.5–38
Duration of employment at the current health institution, years	—	16 (10–22)	0.3–38
**Shift work**			
No	20 (13.2)	—	—
Yes	131 (86.8)	—	—
**Average daily working hours**			
8 h	17 (11.3)	—	—
>8–12 h	99 (65.6)	—	—
>12 h	35 (23.2)	—	—
**Self-rated household material status**			
Good or very good	91 (60.3)	—	—
Average	55 (36.4)	—	—
Poor or very poor	5 (3.3)	—	—
**Presence of chronic disease**			
No	100 (66.2)	—	—
Yes	51 (33.8)	—	—
**Self-rated health**			
Excellent	30 (19.9)	—	—
Very good	54 (35.8)	—	—
Good	30 (19.9)	—	—
Fair	31 (20.5)	—	—
Poor	6 (4.0)	—	—

Data are presented as *n* (%) or median (interquartile range), as appropriate. Ranges indicate the minimum and maximum observed values. Percentages may not total 100.0 because of rounding. IQR, interquartile range.

**Table 2:** Descriptive statistics and internal consistency of the MBI-HSS dimensions (*N* = 151).

MBI-HSS Dimension	Median (IQR)	Mean ± SD	Observed Range	Cronbach’s α
Emotional exhaustion	22 (11–36)	23.93 ± 15.19	0–51	0.940
Depersonalisation	7 (1–11)	7.91 ± 7.09	0–23	0.825
Reduced personal accomplishment	12 (7–18)	12.98 ± 7.41	0–30	0.655

MBI-HSS, Maslach Burnout Inventory–Human Services Survey; IQR, interquartile range; SD, standard deviation. Emotional exhaustion comprised nine items, depersonalisation five items, and reduced personal accomplishment eight reverse-scored items. Higher scores on all three dimensions indicate a less favourable burnout profile.

**Table 3:** Burnout dimensions according to self-rated health category (*N* = 151).

Self-Rated Health	*n*	Emotional Exhaustion, Median (IQR)	Depersonalisation, Median (IQR)	Reduced Personal Accomplishment, Median (IQR)
Excellent/very good	84	17 (9–33.5)	6 (1–10)	12 (7–17)
Good	30	30 (14.75–38)	9 (1–10.5)	12.5 (7–19.25)
Fair/poor	37	33 (14–43)	9 (2–19.5)	12 (9–21.5)
Overall *p* value		0.005	0.272	0.507

Data are presented as median (interquartile range). Overall differences were examined using the Kruskal–Wallis test. Higher scores indicate a less favourable burnout profile. IQR, interquartile range.

**Table 4:** Associations of burnout dimensions with less favourable self-rated health in ordinal logistic regression models (*N* = 151).

Burnout Dimension, per 10-Point Increase	Unadjusted OR (95% CI)	*p*	Adjusted OR (95% CI)	*p*	Mutually Adjusted OR (95% CI)	*p*
Emotional exhaustion	1.44 (1.16–1.78)	0.001	1.31 (1.04–1.64)	0.020	1.30 (0.96–1.75)	0.087
Depersonalisation	1.59 (1.03–2.45)	0.037	1.44 (0.91–2.29)	0.118	1.03 (0.55–1.94)	0.930
Reduced personal accomplishment	1.34 (0.89–2.03)	0.164	1.12 (0.72–1.76)	0.609	0.98 (0.61–1.59)	0.942

The outcome was ordered as excellent/very good, good, and fair/poor self-rated health; therefore, ORs greater than 1 indicate higher odds of being in a less favourable self-rated health category. Each burnout dimension was examined separately in the unadjusted and adjusted models. Adjusted models included age, sex, professional qualification, and chronic disease. The mutually adjusted model additionally included all three burnout dimensions simultaneously. ORs for burnout dimensions are reported per 10-point increase. CI, confidence interval; OR, odds ratio.

**Table 5:** Multicollinearity diagnostics for predictors included in the mutually adjusted ordinal logistic regression model.

Predictor	Tolerance	VIF
Emotional exhaustion	0.518	1.931
Depersonalisation	0.530	1.887
Reduced personal accomplishment	0.846	1.182
Age	0.568	1.759
Sex	0.893	1.120
Professional qualification	0.625	1.599
Chronic disease	0.838	1.193

All VIF values were below 5, and all tolerance values were above 0.20. VIF, variance inflation factor.

**Table 6:** Sensitivity analysis of associations between burnout dimensions and fair/poor self-rated health (*N* = 151).

Burnout Dimension, per 10-Point Increase	Unadjusted OR (95% CI)	*p*	Adjusted OR (95% CI)	*p*
Emotional exhaustion	1.41 (1.09–1.82)	0.008	1.24 (0.95–1.63)	0.118
Depersonalisation	1.63 (0.98–2.73)	0.060	1.51 (0.86–2.63)	0.149
Reduced personal accomplishment	1.40 (0.86–2.30)	0.177	1.22 (0.70–2.11)	0.483

The outcome was fair/poor versus excellent/very good/good self-rated health. Each burnout dimension was examined in a separate binary logistic regression model. Adjusted models included age, sex, professional qualification, and chronic disease. ORs for burnout dimensions are reported per 10-point increase. CI, confidence interval; OR, odds ratio.

**Table 7:** Self-rated health according to shift work and average daily working hours (*N* = 151).

Work-Schedule Characteristic	Excellent/Very Good, *n* (%)	Good, *n* (%)	Fair/Poor, *n* (%)	OR for Less Favourable SRH (95% CI)	*p*
**Shift work**					
No	10 (50.0)	4 (20.0)	6 (30.0)	Reference	
Yes	74 (56.5)	26 (19.8)	31 (23.7)	0.75 (0.31–1.83)	0.532
**Average daily** **working hours**					
8 h	9 (52.9)	4 (23.5)	4 (23.5)	Reference	
>8–12 h	54 (54.5)	23 (23.2)	22 (22.2)	0.94 (0.35–2.52)	0.898
>12 h	21 (60.0)	3 (8.6)	11 (31.4)	0.94 (0.31–2.85)	0.906

Percentages are calculated within each work-schedule category. Odds ratios were estimated using separate unadjusted ordinal logistic regression models. The outcome was ordered as excellent/very good, good, and fair/poor self-rated health; therefore, ORs greater than 1 indicate higher odds of being in a less favourable SRH category. Overall model *p* values were derived from likelihood-ratio chi-square tests and were 0.536 for shift work and 0.991 for average daily working hours. The tests of parallel lines provided no evidence of departure from the proportional-odds assumption for either model (Table 8). CI, confidence interval; OR, odds ratio.

**Table 8:** Tests of the proportional-odds assumption for the ordinal logistic regression models (*N* = 151).

Model	χ^2^	df	*p* Value
Emotional exhaustion—unadjusted	0.012	1	0.912
Emotional exhaustion—adjusted	3.533	5	0.618
Depersonalisation—unadjusted	0.021	1	0.886
Depersonalisation—adjusted	3.340	5	0.648
Reduced personal accomplishment—unadjusted	0.077	1	0.782
Reduced personal accomplishment—adjusted	3.552	5	0.616
All three burnout dimensions—mutually adjusted	3.904	7	0.791
Shift work—unadjusted	0.021	1	0.884
Average daily working hours—unadjusted	4.501	2	0.105

The proportional-odds assumption was evaluated using the test of parallel lines. A non-significant result indicates that there was no evidence of departure from the proportional-odds assumption. Adjusted models for individual burnout dimensions included age, sex, professional qualification, and chronic disease. The mutually adjusted model included all three burnout dimensions together with the same covariates.

## Data Availability

The data supporting the findings of this study are not publicly available because they contain potentially identifiable occupational, sociodemographic, and health-related information collected from participants employed at a single institution. De-identified data may be made available by the corresponding author upon reasonable and justified request, subject to prior approval by the participating institution and, where applicable, the relevant ethics committee, in accordance with applicable ethical, institutional, and data protection requirements.

## Abbreviations

The following abbreviations are used in this manuscript:

CI	Confidence interval
DP	Depersonalisation
EE	Emotional exhaustion
IQR	Interquartile range
MBI-HSS	Maslach Burnout Inventory–Human Services Survey
OR	Odds ratio
PA	Personal accomplishment
RPA	Reduced personal accomplishment
SF-36	36-Item Short Form Health Survey
SRH	Self-rated health
EMS	Emergency medical services
SD	Standard deviation
VIF	Variance inflation factor
df	Degrees of freedom

## Appendix A. Supplementary Details on Outcome Classification and Regression Models

The supplementary tables describe the classification of self-rated health (SRH), compare adjusted estimates across the ordinal and binary models, and summarise work-schedule distributions using the binary health grouping. All tables relate to the same sample of 151 physicians.

**Table A1:** Mapping of the five original self-rated-health responses to the analytical categories.

Original Response	*n* (%)	Primary Ordinal Grouping *n* (%)	Binary Sensitivity Grouping *n* (%)
Excellent	30 (19.9)	Excellent/very good 84 (55.6)	Excellent/very good/good 114 (75.5)
Very good	54 (35.8)		
Good	30 (19.9)	Good 30 (19.9)	
Fair	31 (20.5)	Fair/poor 37 (24.5)	Fair/poor 37 (24.5)
Poor	6 (4.0)		

Percentages use *N* = 151 and may not sum to 100 because of rounding. Counts are derived from the original response frequencies in Table 1. The primary ordinal outcome is ordered from excellent/very good to good and fair/poor. In the binary sensitivity models, fair/poor health is the outcome category of interest; excellent/very good/good health is the reference category.

**Table A2:** Comparison of adjusted associations between burnout dimensions and self-rated health.

Burnout Dimension	Separate Ordinal Models OR (95% CI); *p*	Mutually Adjusted Ordinal Model OR (95% CI); *p*	Separate Binary Models OR (95% CI); *p*
Emotional exhaustion	1.31 (1.04–1.64) *p* = 0.020	1.30 (0.96–1.75) *p* = 0.087	1.24 (0.95–1.63) *p* = 0.118
Depersonalisation	1.44 (0.91–2.29) *p* = 0.118	1.03 (0.55–1.94) *p* = 0.930	1.51 (0.86–2.63) *p* = 0.149
Reduced personal accomplishment	1.12 (0.72–1.76) *p* = 0.609	0.98 (0.61–1.59) *p* = 0.942	1.22 (0.70–2.11) *p* = 0.483

All odds ratios (ORs) are per 10-point increase in the respective burnout score and are adjusted for age, sex, professional qualification, and chronic disease. Separate models include one burnout dimension; the mutually adjusted ordinal model includes all three. Age is continuous; reference categories are female sex, a basic academic degree, and no chronic disease. Ordinal ORs above 1 indicate higher odds of a less favourable SRH category; binary ORs above 1 indicate higher odds of fair/poor SRH. Estimates are reproduced from Table 4 and Table 6. CI, confidence interval.

**Table A3:** Descriptive distribution of binary self-rated health by work-schedule characteristics.

Work-Schedule Characteristic	*n*	Excellent/Very Good/Good *n* (%)	Fair/Poor *n* (%)
**Shift work**			
No	20	14 (70.0)	6 (30.0)
Yes	131	100 (76.3)	31 (23.7)
**Average daily working hours**			
8 h	17	13 (76.5)	4 (23.5)
>8–12 h	99	77 (77.8)	22 (22.2)
>12 h	35	24 (68.6)	11 (31.4)

Percentages are calculated within each work-schedule category. Counts for excellent/very good/good health combine the excellent/very good and good categories reported in Table 7; fair/poor counts are unchanged. The two work-schedule characteristics each describe the full sample of 151 physicians. This table is descriptive; the ordinal regression analyses of work schedule are reported in Table 7.
